# The one-step fabrication of porous hASC-laden GelMa constructs using a handheld printing system

**DOI:** 10.1038/s41536-023-00307-1

**Published:** 2023-06-10

**Authors:** SeoYul Jo, JiUn Lee, Hyeongjin Lee, Dongryeol Ryu, GeunHyung Kim

**Affiliations:** 1 Department of Precision Medicine, Sungkyunkwan University School of Medicine, Suwon, Republic of Korea; 2 Department of Biotechnology and Bioinformatics, Korea University, Sejong, Republic of Korea; 3 Department of Biomedical Science and Engineering, Gwangju Institute of Science and Technology, Gwangju, Republic of Korea; 4 Department of Biophysics, Institute of Quantum Biophysics, Sungkyunkwan University, Suwon, Republic of Korea

**Keywords:** Tissue engineering, Biomedical materials

## Abstract

The fabrication of highly porous cell-loaded structures in tissue engineering applications has been a challenging issue because non-porous cell-laden struts can cause severe cell necrosis in the middle region owing to poor transport of nutrients and oxygen. In this study, we propose a versatile handheld 3D printer for the effective fabrication of porous cell-laden methacrylated gelatin (GelMa) with high porosity (≈97%) by air injection and a bubble-making system using mesh filters through which a mixture of air/GelMa bioink is passed. In particular, the pore size and foamability of the cell constructs could be manipulated using various processing parameters (rheological properties of GelMa, filter size and number, and air-bioink volume ratio). To demonstrate the feasibility of the cell construct as a tissue engineering substitute for muscle regeneration, in vitro cellular activities and in vivo regeneration ability of human adipose stem cells were assessed. The in vitro results demonstrated that the human adipose stem cells (hASCs) fabricated using the handheld 3D printer were alive and well-proliferated. Furthermore, the in vivo results showed that the hASCs-constructs directly printed from the handheld 3D printer showed significant restoration of functionality and efficient muscle regeneration in the volumetric muscle loss model of mice. Based on these results, the fabrication method of the porous cell-laden construct could be a promising tool for regenerating muscle tissues.

## Introduction

Bioengineered tissue substitutes should be highly porous, biocompatible, biodegradable, and mechanically stable to facilitate various cellular activities, including cell growth and differentiation^1^. Recently, cell-encapsulating methods that incorporate single or multiple cells during scaffold-fabricating processes, such as 3D printing, microfluidic, and photolithography, have been actively developed because it is much easier to efficiently position multiple cells and provide a more suitable microenvironment compared to conventional cell-seeding scaffolds^2–4^. In particular, 3D bioprinting uses various bioinks consisting of cells, growth factors, and several polysaccharides or protein-based matrix-hydrogels, and by using a multi-layered printing approach supplemented with mechanical or electrical systems, tissue-engineering cell-laden 3D constructs can be attained successfully. Eventually, the bioprinted cell constructs could be transferred to various bioreactors that guide the growth of tissue engineering structures or directly transplanted into damaged tissue regions.

However, like problems that occur in most cell constructs manufactured using cell-encapsulating processes, the cell constructs fabricated using a 3D bioprinting process also have some overcoming issues. In volumetric cell-laden hydrogel constructs, non-porous thick cell-laden struts can cause severe cell necrosis in the middle region owing to poor transport of nutrients and oxygen^5–7^. For example, cell-laden hydrogel struts (3.5 wt% of alginate) thicker than 200 μm in diameter cannot provide satisfactory paths to transport various nutrients and remove waste, resulting in cell necrosis in the center region of the cell-printed alginate struts^8^. Furthermore, the viability of cells laden in porous alginate constructs was significantly higher (approximately 31% increase) than that of the non-porous cell-laden constructs^9^.

In particular, non-porous cell-laden hydrogels can provide a low degree of penetration of nearby blood vessels into the cell blocks. Therefore, many researchers have attempted to obtain porous cell-laden constructs (Table 1)^10–16^. Therefore, the design and fabrication of an exceptional bioink for fabricating porous cell-laden constructs is challenging. The most widely used method to prepare cell-laden porous constructs is the use of non-toxic sacrificial beads. Recently, gelatin gel beads have been used as sacrificial materials to fabricate porous, cell-laden scaffolds^10^. The gelatin beads were mixed with a cell-laden sodium alginate solution, and the mixed bioink was then immersed in a calcium chloride solution. Eventually, a porous cell-laden alginate scaffold was obtained by leaching the gelatin beads at 37 °C. The process can be highly impactive because no toxic chemicals are used in this process, and the viability of laden cells is significantly high, indicating that the process is safe. However, the porosity of the fabricated cell construct was limited to approximately 70%, and a homogeneous distribution of gelatin beads is required to obtain a porous construct. Furthermore, protein-based bioinks have been used to fabricate highly porous cell-laden constructs using the concept of whipped cream production^11,12^. The whipped proteins were mixed with cells, and the bioink was loaded for 3D bioprinting. Through in vitro and in vivo results, the formed bioink showed much greater cellular activity and vascularization compared to those of non-porous cell-laden hydrogel constructs^11^. Porous cell constructs using the whipping method, however, require a particular surfactant such as poly(vinyl alcohol)^11^. A vigorous whipping procedure is required in which stirring conditions (solution concentration, stirring time, stirring speed, etc.) can be optimally selected because the generated air-bubble diameter and air volume fraction can be directly affected by the whipping condition^11,12^. In addition, a careful additional mixing process of the whipped solution and cells to obtain highly porous and homogenous cell-laden structures should be essential^11,12^. For these reasons, the whipping method does not allow for realistic in situ fabrication (i.e., simultaneous generation of air bubbles and homogeneous cell mixing) of highly porous cell-laden constructs. Recently, a simple air injection method through a syringe was used to enable the fabrication of GelMA foam containing human adipose stem cells (hASCs)^13^. However, owing to a complicated process using two-step procedures (air injection and cell encapsulation), the control of pore sizes was challenging. Furthermore, various methods including aqueous two-phase emulsion (ATPE)^14^, gas foaming^15^, and microfluidic systems^16^ have been implemented to obtain highly porous cell-laden constructs, but these approaches were not easy versatile and fully efficient due to a complex process or sacrificing materials (Table 1).

**Table 1:** Fabrication of the porous cell-laden constructs using various foaming processes.

Methods	Cell types (density)	Hydrogel	Cross-linking condition	Pore size	Advantages	Disadvantages
Sacrificial porogen^10^	Liver hepatocellular carcinoma cell line (HepG2, 5 × 10^6^ cells mL^−1^)	Alginate	Agarose with 2 w/v % CaCl_2_	150~300 μm	- Enabling 3D encapsulation of cells - Simultaneous controlling of porosity	- Separated three-step processes of preparing gelatin microsphere, mixing, and crosslinking
Whipping^11^	Murine myoblast cell line (C2C12, 3 × 10^6^ cells mL^−1^)	GelMa, PVA with LAP solution	UV irradiation (20 W, 1 min)	50~400 μm	-Multiscale and interconnected porous structure -Broad adaptability for tissue engineering applications (3D bioprinting process)	- Separated two-step processes of whipping and cell encapsulation - Addition of surfactant required to increase foam stability
Whipping^12^	Human derived adipose stem cells (hASCs, 1 × 10^6^ cells mL^−1^)	Collagen	1 mM Genipin	≤400 μm	- Sacrificial material not used - Homogeneous distribution of cells - Broad adaptability for tissue engineering applications (3D bioprinting and injectable process)	- Separated two-step processes of whipping and cell encapsulation - Careful mixing process - Time consuming for whipping process
Air injection^13^	Human derived adipose stem cells (hASCs, 2 × 10^6^ cells mL^−1^)	GelMa with LAP solution	UV irradiation (4 W cm^−2^ for 2 s)	430~490 μm	- Homogeneous viable cell distribution throughout the whole depth of biomaterials	- Difficulty in controlling the range of pore sizes and distribution - Separated two-step processes of air injection and cell encapsulation
Aqueous two-phase emulsion (ATPE)^14^	Mouse fibroblasts cell line (NIH/3T3) and HepG2 (1 × 10^6^ cells mL^−1^)	GelMa with LAP solution	UV irradiation (0.5 W cm^−2^ for 30 s)	≤100 μm	- Widely adjustable micropore size - Broad adaptability for bioprinting process (extrusion and DLP modalities)	- Requires multiple washing steps to remove sacrificial polymers
Gas foaming with H_2_^15^	Rat bone marrow stem cells (rBMSCs, 1 × 10^7^ cells mL^−1^)	Gelatin, sodium alginate with NaCl solution	0.1 M CaCl_2_ (25 °C for 30 min)	125~250 μm	- Adaptability for injectable hydrogel - Interconnected pores formed via simple and cost-effective method	- Time consuming crosslinking process required
Microfluidic system with N_2_^16^	Human non-small-cell lung cancer cell line (A549, 1.2 × 10^5^ cells mL^−1^)	Gelatin with pluronic F-127	1.2 mg ml^−1^ Microbial transglutaminase (mTGase)	114 μm	- Homogeneous distribution of cells and pore size	- Separate controlling of three inlets - Difficulty in controlling the range of pore sizes

In this work, we propose a new method to obtain highly porous human adipose stem cell (hASCs)-laden constructs with above 90% porosity without the complex and time-consuming preparation of a porous bioink. To achieve this goal, the one-step printing system was supplemented with three microscale mesh filters to entrap air bubbles in the bioink. GelMa was used as the matrix hydrogel as an air-bubble-cell-laden bioink. This biofabrication has two advantages compared to previous whipping/printing methods: (1) the printing system only requires cell-laden GelMa solution (does not require a separately prepared porous hydrogel solution) and clean air, which can permit instantaneous mixing of cells and air during the printing; and (2) the process doesn’t require a complex and heavy conventional 3D printer, so that a light handheld 3D printer can be easily and effectively used to deposit the porous cell-laden construct in any defective tissue region in a short time.

To choose the appropriate air bubble formation of the cell-loaded GelMa solution using a handheld 3D printer, the effect of microscale mesh filter size and number of filters on the air bubble foamability and pore size was evaluated. In addition, to show the biological activities of the porous cell-laden constructs, in vitro cellular activities were assessed, and we further examined the feasibility of using the porous GelMa constructs with hASCs, which were directly obtained using a handheld 3D printer, for the functional restoration of volumetric muscle loss in mice.

## Results and discussion

Recently, bioprinted cell-laden structures have been extensively researched in various tissue engineering applications owing to their ability to distribute cells homogeneously within a cell structure and to deposit numerous cells in a required region of cell constructs^17,18^. However, bioprinted cell-laden constructs have a shortcoming, that is, relatively poor metabolic activity due to the cross-linked non-porous hydrogel cell-block with relatively low porosity, although the precise and well-controlled degradation rate of hydrogels can handle this problem to some extent. Thus, designing a highly porous cell-laden construct is a key factor for efficient tissue regeneration because the porous structure can directly affect the successful vascularization and comfortable transport of various nutrients and waste products after implantation^19,20^. From this reason, we designed a new in situ fabrication method for a cell-laden porous structure using an air-bubbling process attached to a handheld 3D printer. GelMa with methacrylation (87.6 ± 0.5%) was used to fabricate porous cell-laden constructs because the hydrogel has been widely considered in various cell-laden structures owing to its resemblance to the extracellular matrix, easy controllability of its rheological and physical properties under processing temperature, and effective crosslinking ability^21–23^.

**Fig. 1:**
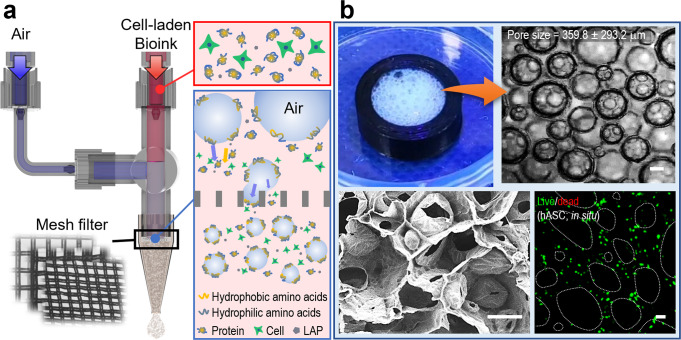
Schematics of an in situ handheld printing system. **a** A schematic of an in situ handheld printing system to obtain microbubbles. **b** Optical and SEM images of a porous hASCs-laden GelMa structure located in a cylindrical mold. Scale bar, 200 µm (**b**).

A schematic of the in situ handheld 3D printer is shown, and the microbubbling mechanism is shown in Fig. 1a. The handheld 3D printer consisted of two channels of air, a cell-laden GelMa bioink, and several microscale mesh filters to efficiently induce the development of microscale air bubbles. Air bubbles in the liquid phase are thermodynamically unstable, so a clear size range of the fabricated air bubbles has not been easily obtained^24,25^. In this system, we used air bubble development *via* air blowing through GelMa bioink. As shown in the simple schematic to form the air bubbles, the air bubbles as a dispersed phase can be accompanied by the air-injection and filtering processes; the discontinuous big air bubbles injected and mixed with GelMa bioink can be deformed and broken through the microscale mesh filters, and amphiphilic GelMa can be moved in the newly generated air-phase boundary and eventually be formed into self-assembled GelMa pores. Figure 1b shows the fabricated porous hASCs-laden GelMa structure in which homogenously distributed cells and microbubbles (pore size = 359.8 ± 293.2 μm) were obtained. In terms of morphology, the pores were hierarchically composed of macropores and micropores within the porous structure, and through the cross-sectional SEM image, well-interconnected pores were obtained. In addition, the cell viability in the porous GelMa construct was approximately 98.3%, indicating that the air bubbling process is safe for laden cells.

### Effect of rheological properties of GelMa bioink on the bubble formation

**Fig. 2:**
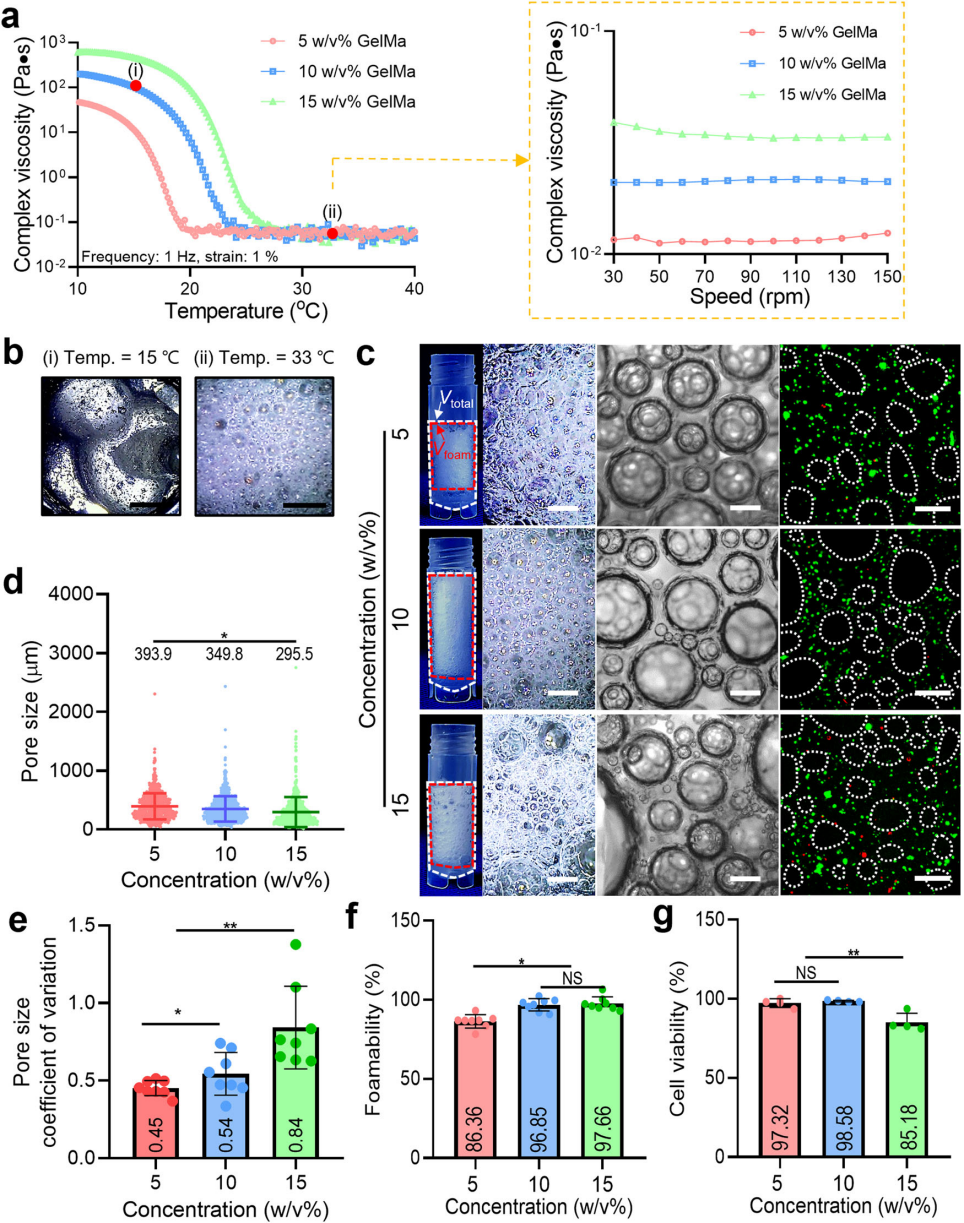
Bubble formation for various rheological properties of bioink. **a** Complex viscosity (Pa·s) of various GelMa concentrations (5, 10, and 15% w/v) and (**b**) the air-bubbles formed at two typical temperatures [(i) 15 °C and (ii) 33 °C]. **c** Optical and live (green)/dead (red) images of air bubbles formed with various GelMa concentrations (V_total_: total volume including air bubbles and solution and V_foam_: volume of air bubbles). **d** Pore size, (**e**) pore size coefficient of variation, defined with standard deviation divided by average value, (**f**) foamability (V_foam_/V_total_), and (**g**) cell viability of the porous GelMa structures for various GelMa concentrations. All data are presented as mean ± standard deviation and the *p*-values were determined by one-way ANOVA followed by Tukey’s test (**d**, **e**, **f**, **g**) (NS = statistical nonsignificance, *p < 0.05, **p < 0.005, and ***p < 0.0005). Scale bar, 2 mm (**b**); 1 mm (b-optical images); 200 µm (b-microscopic images and live/dead images).

To observe the effect of viscosity on air bubble formation and cell viability, we measured the complex viscosity (Pa·s) of three different GelMa solutions (5, 10, and 15% w/v) showing a typical sol-gel transition region (Fig. 2a). Based on these results, two typical temperatures (gel region at 15 °C and sol region at 33 °C) were selected to evaluate air bubble formation, as shown in Fig. 2a. As shown in Fig. 2b(i) and (ii), at a low temperature (15 °C) of the GelMa solutions, the air bubbles were significantly unstable compared to the relatively higher temperature (33 °C). This phenomenon occurs because the large air bubbles in the high-viscosity GelMa solution (at 15 °C) before passing through the mesh filters of the handheld 3D printer can maintain their large size owing to the high viscosity and surface tension even after passing through the filters, whereas the large air bubbles in the relatively low-viscosity GelMa solution (at 33 °C) can break up into much smaller air bubbles when passing through the filters, as shown in the optical images of Fig. 2b(i) and (ii).

In addition, to evaluate the effect of GelMa solution concentration on the formation of air bubbles, three different concentrations (5, 10, and 15% w/v) were used. In the test, the processing temperature was set at 33 °C, and three mesh filters (pore size = 121.2 ± 43.9 μm) and volume ratio (1:3) of the bioink and air were used with 3 mL/s flow rate. As shown in the optical images (Fig. 2c) and quantitative analyses (Fig. 2d–e and Supplementary Fig. 1) of the pore size and size distribution, the 15% w/v GelMa bioink showed a significantly wider size distribution of air bubbles compared to the lower GelMa concentrations, which may be due to the slightly higher viscosity and surface tension compared to those of 5 and 10% w/v GelMa.

In addition, the foam generation capacity can be measured through foamability^26^. In terms of foamability (%), which is calculated using ‘foam volume (V_foam_)/total volume (V_total_) × 100’ for the fabricated pore structure, the concentration (5% w/v) of GelMa showed significantly poor foamability because of the insufficient polar group of the GelMa solution (Fig. 2f). In addition, the cell viability of C2C12 cells measured to be 97.32, 98.58, and 85.18% for porous GelMa structures fabricated using 5, 10, and 15% w/v GelMa, respectively (Fig. 2g). We attribute this phenomenon to the increased viscosity of the concentrated GelMa bioink inducing considerable shear stress during the foaming process^27,28^. From the results of air-bubble formation and cell viability caused by the processing temperature and GelMa concentration, 10% w/v GelMa and 33 °C processing temperature were chosen for this process.

**Fig. 3:**
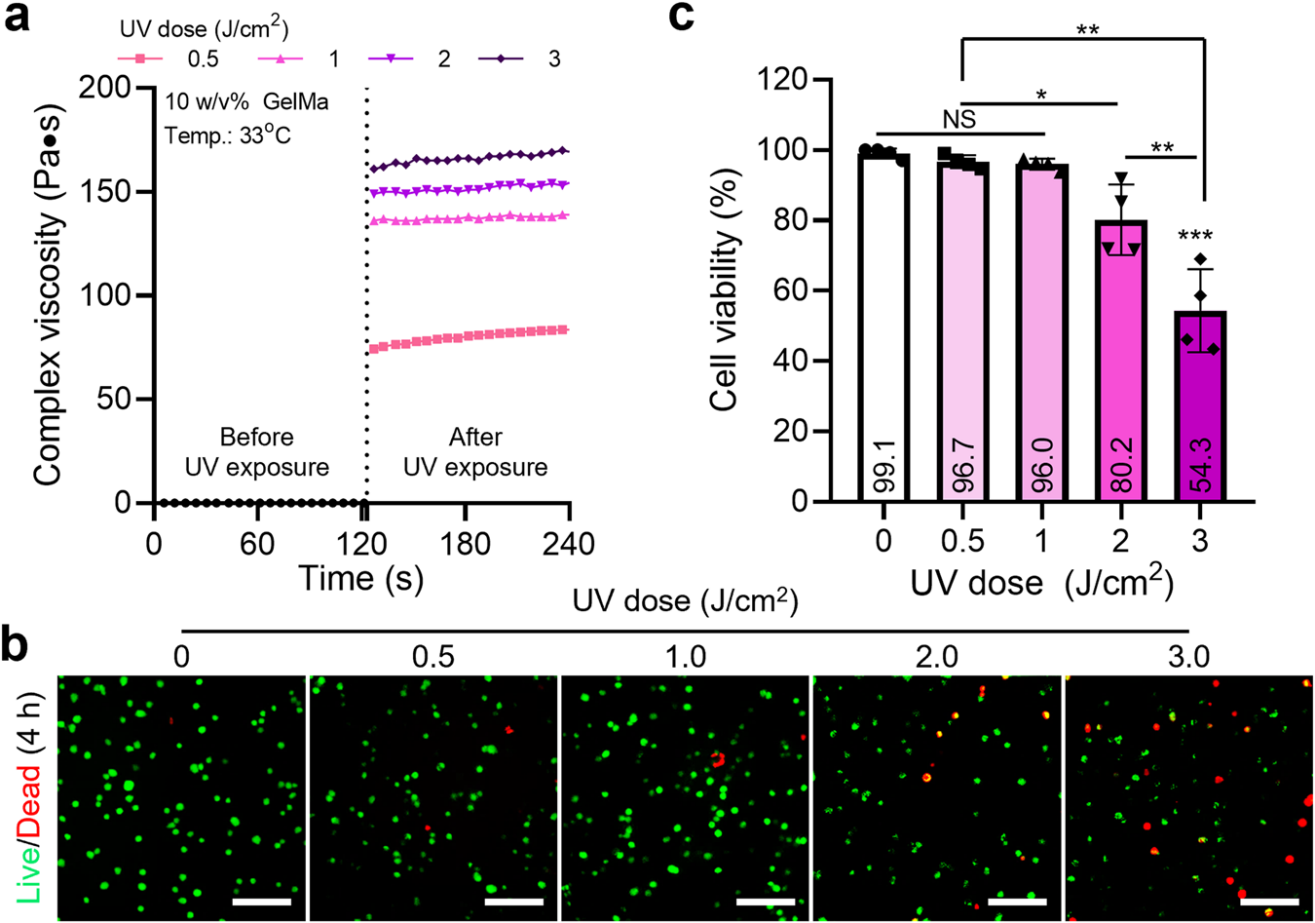
Cell viability for various UV doses. **a** Complex viscosity (Pa·s) before and after UV exposures for the 10% w/v GelMa solution. **b** Live (green)/dead (red) images and (**c**) cell viability (%) for various UV doses (0–3 J/cm^2^). All data are presented as mean ± standard deviation and the *p*-values were determined by one-way ANOVA followed by Tukey’s test (**c**) (NS = statistical nonsignificance, *p < 0.05, **p < 0.005^,^ and ***p < 0.0005). Scale bar, 200 µm (**b**).

To evaluate the crosslinking ability of the porous GelMa structure for various UV doses (J/cm^2^), the complex viscosity of the GelMa bioink (10% w/v) was observed at several UV intensities (Fig. 3a). Evidently, crosslinking of the GelMa bioink was performed well at the UV doses. In general, the cell viability of cell-laden methacrylated hydrogels can be closely related to the photo-crosslinking condition, so the cell viability of the porous cell-laden GelMa form that was fabricated using the previously fixed processing condition was measured. As shown in the live (green)/dead (red) images in Fig. 3b and their cell viability (Fig. 3c), the cell viability of the porous GelMa construct was approximately 90% below the UV dose (1 J/cm^2^); however, as the UV power increased, the cell viability gradually decreased, demonstrating that the increased UV dose caused significant damage to the cells laden in the porous structure.

### Effects of filter size and number attached to the handheld 3D printer on air bubble formation

**Fig. 4:**
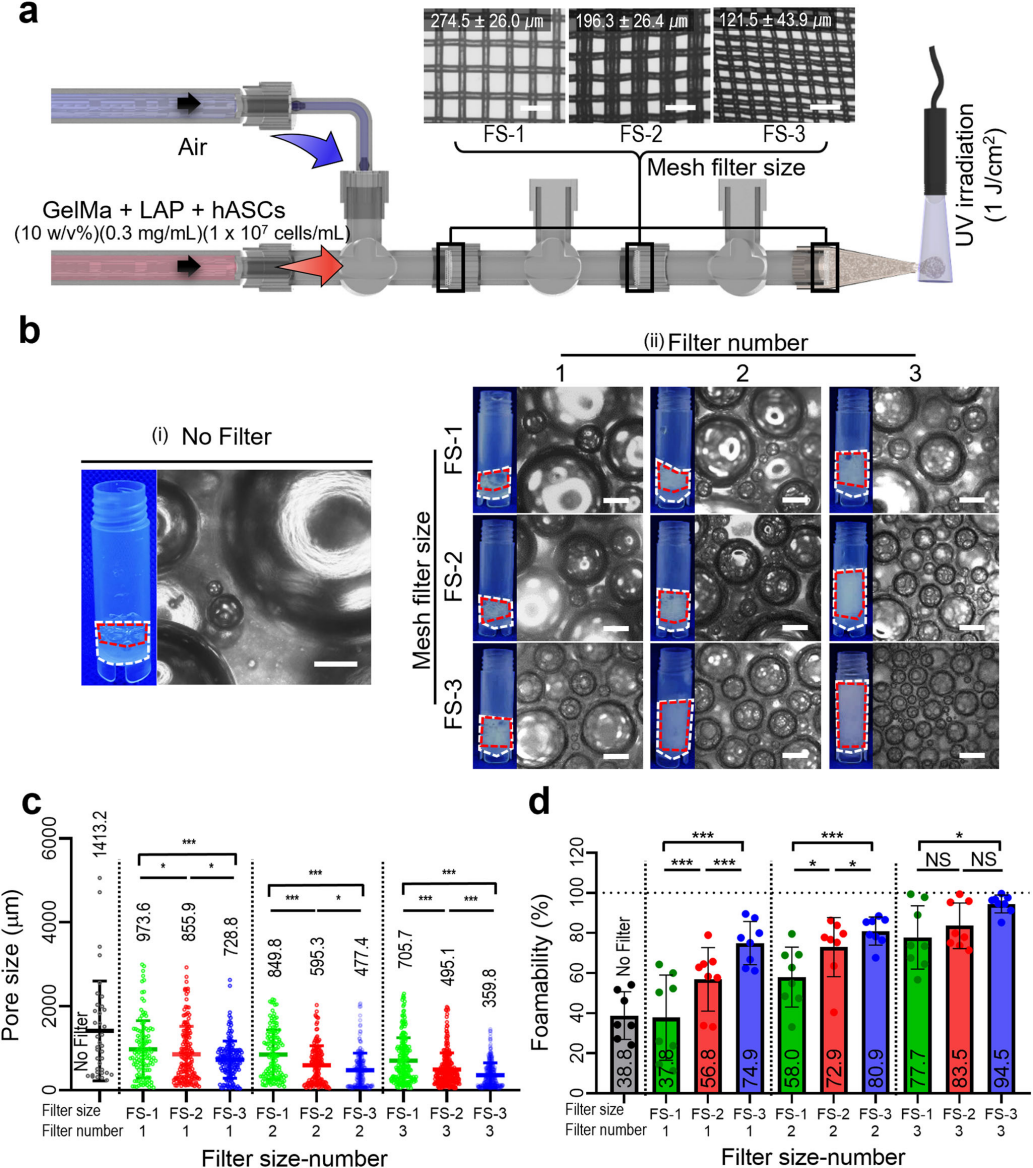
Bubble formation for various mesh filter conditions. **a** Schematic of a handheld printing system with various sizes of mesh filter. **b** Optical images of the air bubbles formed with various sizes and numbers of the mesh filter [(i) without filter and (ii) with filters]. **c** Fabricated pore size and (**d**) foamability of the porous GelMa structures for various filter sizes and filter numbers. All data are presented as mean ± standard deviation and the *p*-values were determined by one-way ANOVA followed by Tukey’s test (**c**, **d**) (NS = statistical nonsignificance, *p < 0.05, **p < 0.005^,^ and ***p < 0.0005). Scale bar, 500 µm (**a**, **b**).

To assess the effect of the three different poly(ethylene) filter sizes [FS-1 (pore size = 274.5 ± 26.0 μm), −2 (196.3 ± 26.4 μm), −3 (121.5 ± 43.9 μm)] and filter number (FN), which were used in the handheld 3D printer shown in Fig. 4a, on the foamability and pore size of the porous GelMa constructs, the volume flow rate and mixing ratio (bioink: air) were set as 3 mL/s and 1:3. The other parameters are described in the schematic in Fig. 4a. As shown in the optical images in Fig. 4b(i), a filter was not inserted in the handheld 3D printer, and significantly large pores (1413.2 ± 1190 μm) appeared. However, when the filters were placed in the handheld 3D printer, the pore size and foamability of the fabricated bioinks were significantly reduced. In particular, the decrease in FS induced a much smaller pore size and efficient development of the foamability of the bioink (Fig. 4c–d and Supplementary Fig. 2). Furthermore, the increase in FN accelerated the pore size reduction and increased the foamability (Fig. 4c–d). However, as the number of FN exceeded ‘3’, the foamability and pore size reduction were saturated, and also when using much smaller filter size, too high pressure was required to flow the cell-laden GelMa bioink through the handheld 3D printer, so we didn’t use a mesh filter with the smaller filter size than that of the FS-3 filter. Based on the results, the three-filter number and filter size (FS-3, 121.5 ± 43.9 μm) were used in the handheld 3D printer.

### Effect of volume ratios of bioink and air on the bubble formation

**Fig. 5:**
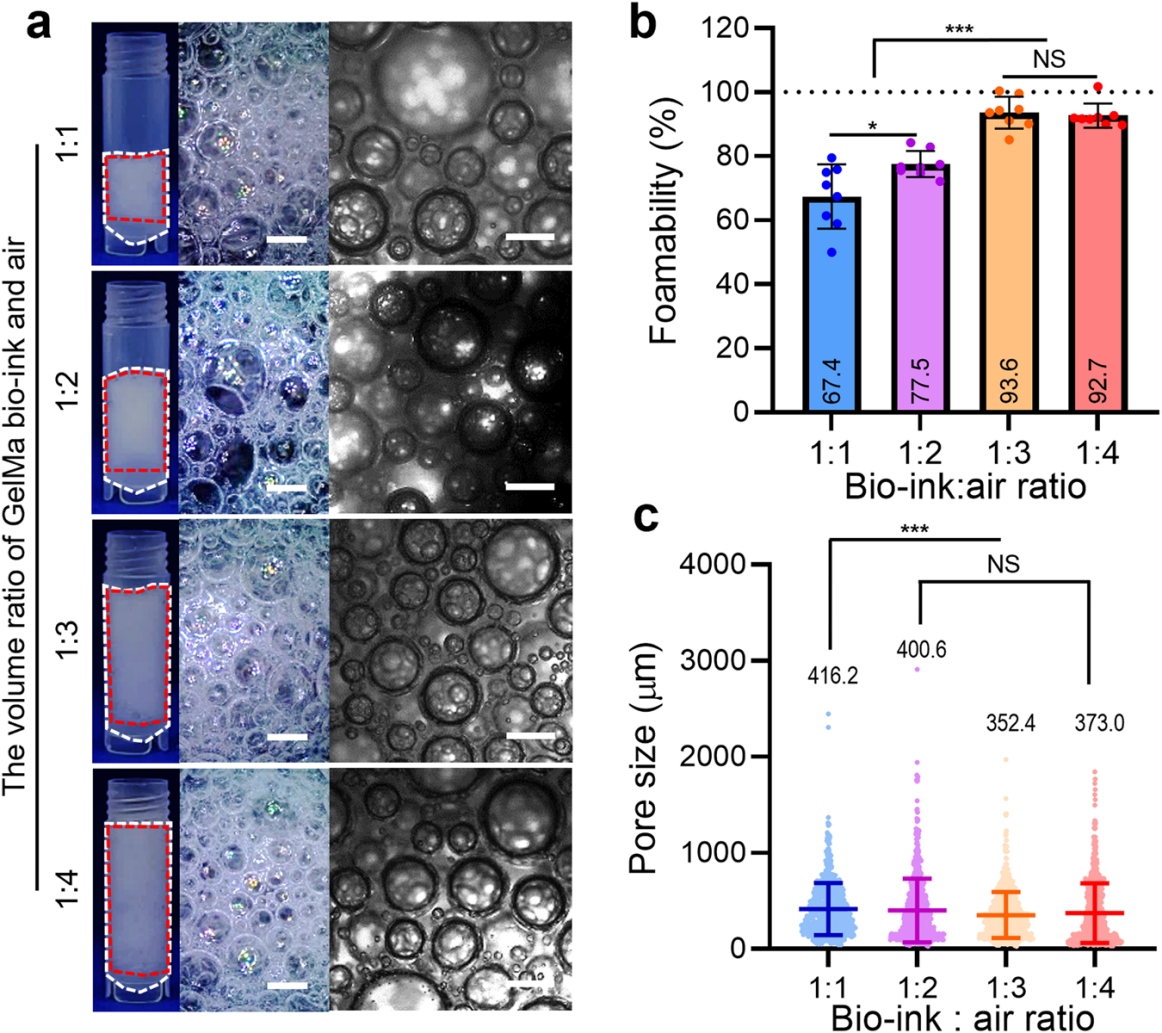
Bubble formation for various volume ratios of bioink and air. **a** Optical images (red dot-line = V_foam_ and white dot-line = V_total_), (**b**) foamability, and (**c**) pore size of the porous GelMa structures fabricated with various volume ratios of bioink and air. All data are presented as mean ± standard deviation and the *p*-values were determined by one-way ANOVA followed by Tukey’s test (**b**, **c**) (NS = statistical nonsignificance, *p < 0.05, **p < 0.005^,^ and ***p < 0.0005). Scale bar, 1 mm (b, a-optical images); 500 µm (b-microscopic images).

Four different mixing ratios of GelMa bioink and air (1:1, 1:2, 1:3, and 1:4) were used to evaluate the effect of the mixing ratio on the pore size and foamability of the fabricated porous GelMa constructs. In Fig. 5a, optical images of the fabricated GelMa constructs attained with various mixing ratios and established processing conditions are shown. As the air volume fraction increased, foamability gradually increased from 67% to 93% (Fig. 5b). However, when the mixing ratio exceeds 1:3, the foamability of the GelMa bioink saturated at approximately 93%. Interestingly, the difference in the pore size for the mixture ratios (1:2–1:4) of bioink and air was nonsignificant (Fig. 5c and Supplementary Fig. 3). From the results, we can observe that the bioink-air mixing ratio directly affects the foamability, not the pore size, of the fabricated GelMa construct. Based on these results, we fixed the bioink-air mixing ratio to 1:3.

### Effects of flow rate on air bubble formation and cell viability

**Fig. 6:**
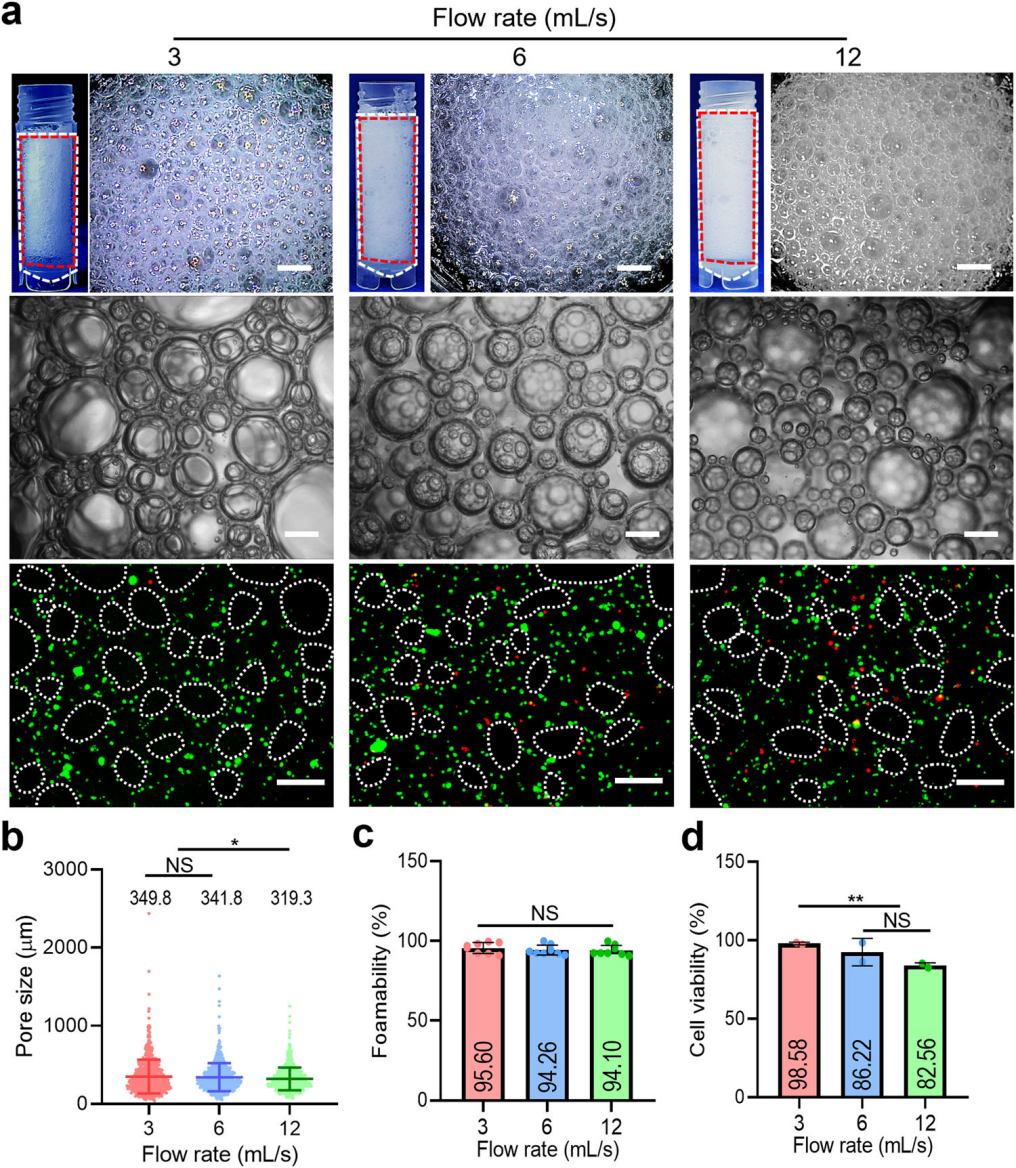
Bubble formation for various flow rates. **a** Optical images and live (green)/dead (red) images of porous GelMa constructs fabricated using various flow rates (3~12 mL/s). Measured (**b**) pore size, (**c**) foamability, and (**d**) cell viability. All data are presented as mean ± standard deviation and the *p*-values were determined by one-way ANOVA followed by Tukey’s test (**b**, **c**, **d**) (NS = statistical nonsignificance, *p < 0.05, **p < 0.005^,^ and ***p < 0.0005). Scale bar, 1 mm (a-optical images); 200 µm (a-microscopic images and live/dead images).

As the flow rate of the bioink is one of major constituents to induce wall shear stress that can affect the viability of cells^28–30^, various flow rates of the C2C12-laden GelMa bioink were used to obtain porous GelMa structures using fixed mesh filter size (FS-3) and mixing ratio of bioink and air (1:3) (Fig. 6a). As result, the pore sizes of porous GelMa structures fabricated using the flow rates (3, 6, and 12 mL/s) were measured to be 349.8, 341.8, and 319.3 μm (Fig. 6b and Supplementary Fig. 4), and the foamability was 95.6, 94.26, and 94.1% (Fig. 6c), respectively. Figure 6d demonstrates the cell viability for the flow rates, and the viability has gradually decreased with the increased flow rate of the GelMa bioink, which can be clearly due to the enhanced wall shear stress. Based on the results, we have fixed the flow rate of the GelMa bioink to 3 mL/s to fabricate the porous GelMa structures.

### In vitro cellular activities

To validate the practicability of the cell-laden porous GelMa constructs, a non-porous cell-laden GelMa hydrogel (5% w/v) and a porous cell-laden GelMa construct obtained using the handheld 3D printer were used as the control and experimental groups, respectively. In both cell-laden GelMa structures, hASCs (density = 1 × 10^7^ cells/mL) were used because they can effectively differentiate into myogenic lineages under biochemical and biophysical cues^31–33^.

**Fig. 7:**
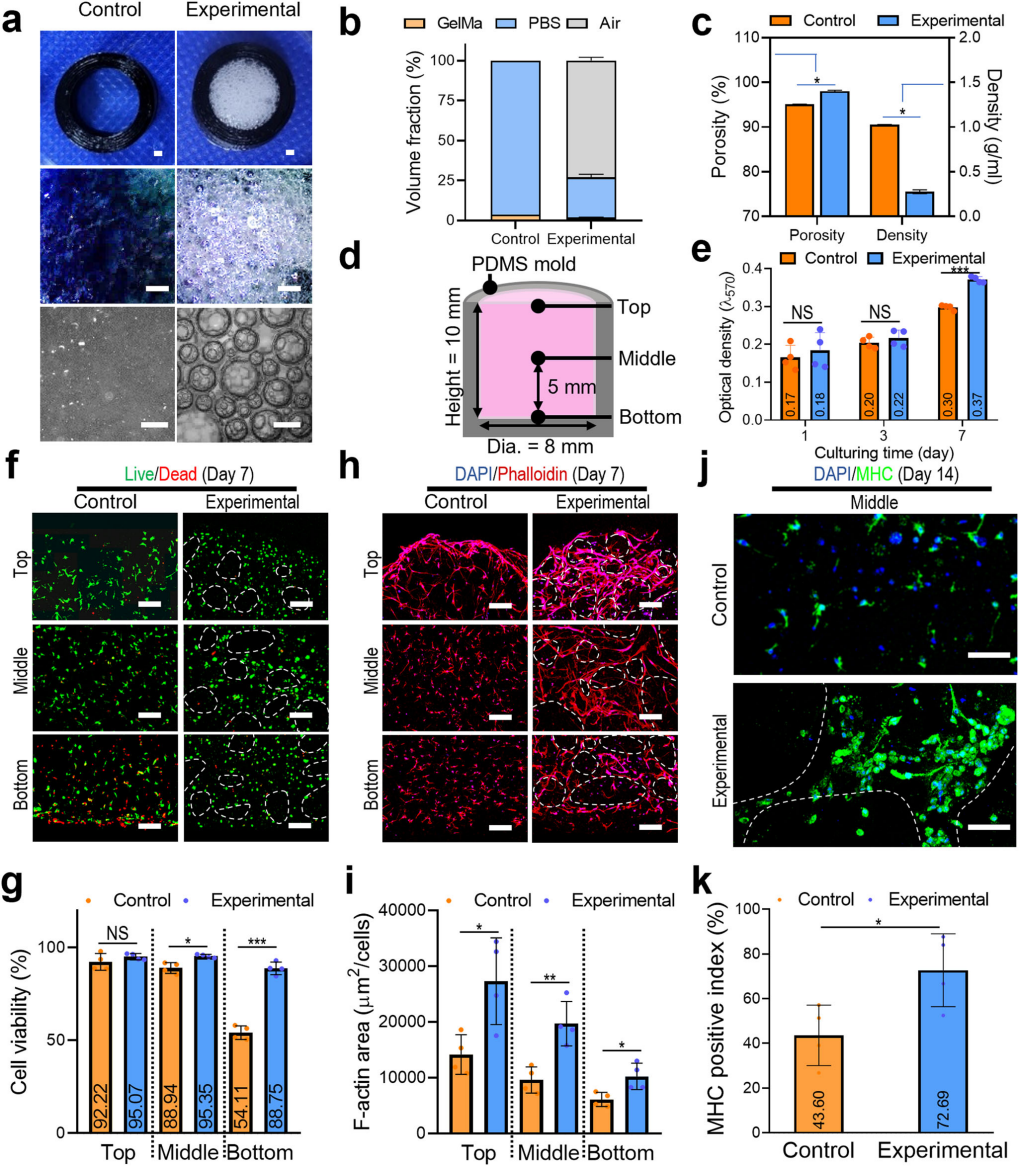
In vitro study of the porous GelMa structure compared to bulk hydrogel. **a** Optical images of the normal non-porous bioink (control) and porous bioink (experimental). **b** Volume fraction of the bioinks consisting of GelMa, PBS, and air of the fabricated structures. **c** Porosity and density of the structures. **d** Schematic of the cell containing mold. **e** MTT assay of hASC proliferation in both structures at 1, 3, and 7 days. **f** Live/dead images at 7 days and (**g**) cell viability. **h** Dapi (blue)/phalloidin (red) images and (**i**) F-actin area. **j** Dapi (blue)/MHC (green) images of the top, middle, and bottom region in the control and experimental group and (**k**) positive index of MHC. All data are presented as mean ± standard deviation and the *p*-values were determined by student’s *t*-test (**c**, **e**, **g**, **i**, **k**) (NS = statistical nonsignificance, *p < 0.05, **p < 0.005^,^ and ***p < 0.0005). Scale bar, 1 mm (a-optical images); 500 µm (b-microscopic images); 200 µm (**f**, **h**); 100 µm (**j**).

The hASC-GelMa bioink (control) was loaded through a normal syringe pump and injected into a mold, and the porous bioink (experimental) that was processed with the handheld 3D printer with the previously established processing parameters was also injected into the same mold (Fig. 7a). The optical images showed well-embedded and interconnected air bubbles (avg. pore size = 359.8 μm), whereas the control sample did not exhibit any porous structure. Quantitative analysis of the volume fraction of the bioink consisting of GelMa, PBS, and air in both constructs showed that the air volume in the experiment was approximately 65%, which is consistent with the designed air volume (bioink: air = 1:3). Furthermore, we measured the porosity and density of the fabricated porous constructs, and significantly higher porosity (97%) and lower density (0.25 g/mL) were observed in the experimental group than in the control group (Fig. 7c).

To further evaluate the cellular activities of the control and experimental bioconstructs filled in the PDMS mold (Fig. 7d), cell proliferation was measured using an MTT [3-(4,5-dimethylthiazol-2-yl)-2,5-diphenyltetrazolium bromide] assay. The cells in control and experimental bioconstructs have proliferated, however, the cells cultured in the experimental bioconstructs showed significantly higher proliferation compared to control (Fig. 7e). These results can be attributed to the higher porosity in the experimental structure^34,35^. Live/dead images of the constructs in the top, middle, and bottom regions were captured on day 7 (Fig. 7f). As shown in the result, the cultured cells in the control group showed significantly lower cell viability in the bottom region (54.11%), whereas in the experimental group, the cell viability in the bottom region was relatively high (88.75%) (Fig. 7g). These results could indicate that the porous structure can effectively transfer nutrients and metabolites.

Previous several works have suggested the importance of F-actin activities, which can modulate various cellular functions including cell locomotion, adhesion, proliferation, and even differentiation^36–38^. As shown in Fig. 7h, nuclei/F-actin of the cells on day 7 in the top, middle, and bottom regions of both the constructs. The results showed that the cytoskeleton of the porous experimental construct was more actively stretched or expanded than that of the control, independent of the top, middle, and bottom regions of the construct. The F-actin activity was quantitatively measured using the F-actin area (μm^2^) per cell of the control and experimental constructs (Fig. 7i). In addition, according to several works, the depolymerization of F-actin can be an indication of necrosis^39,40^. Based on the meaningful development of F-actin on hASCs cultured in the experimental bioconstructs compared to the control group, we carefully predict that the porous architectures can prevent necrotic region.

The myogenic activities of the hASCs in control and experimental bioconstructs were evaluated using MHC immunofluorescent images shown in Fig. 7j. As a result, the MHC positive index of the experimental bioconstructs was significantly higher compared to the control (Fig. 7k). From the in vitro results, we can conclude that the experimental construct can provide a more promising cellular environment for the laden cells to evoke high cellular activity compared to the control, normal hydrogel bioink. This can be attributed to the porous structure induced by efficient cell-to-cell interactions and effective transport of nutrients and metabolic waste^5–7^.

### Handheld printing system and its application

**Fig. 8:**
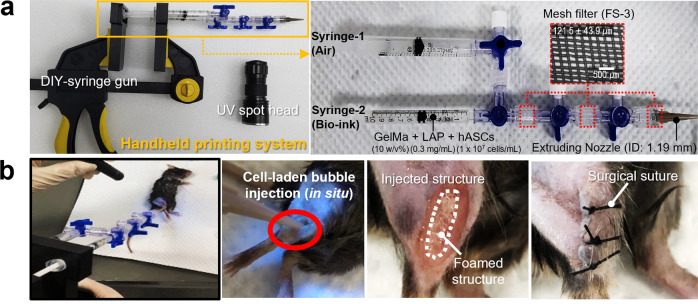
Application of the handheld printing system. Optical images of (**a**) the handheld printing system and its application on (**b**) a mouse VML model.

The in situ printing process refers to the simultaneous fabrication and implantation of bioconstucts to the defect site, whereas, ex situ printing process requires a separate fabrication and implantation process^41^. As such, in a time-sensitive emergency, in situ fabrication process would be a much more appropriate application compared to the latter^42^. From the aspect, the in situ printing process enables the direct transfer of biomaterials to the patient, which eliminates numerous issues associated with ex vivo printing. Moreover, the in situ bioprinted structures may exhibit superior functionality and integration compared to ex vivo printed implants, as they benefit from the natural cellular microenvironment of the body^43^. To demonstrate the in situ printing ability of the air-bubble-laden bioink using the handheld 3D printing system, two models used a mouse VML. Figure 8a shows the handmade 3D printer, which consists of a mixing channel of air and cell-laden GelMa bioink and three mesh filters with size (FS-3). By using the handheld 3D printer, we directly printed the porous cell-laden GelMa bioink on the defective region, and simultaneously applied UV intensity (UV dose = 1 J/cm^2^) to the printing area. As shown in the result, the air-bubbled GelMa structure was well covered in the defected region ≈2 s (Fig. 8b).

### In vivo studies

**Fig. 9:**
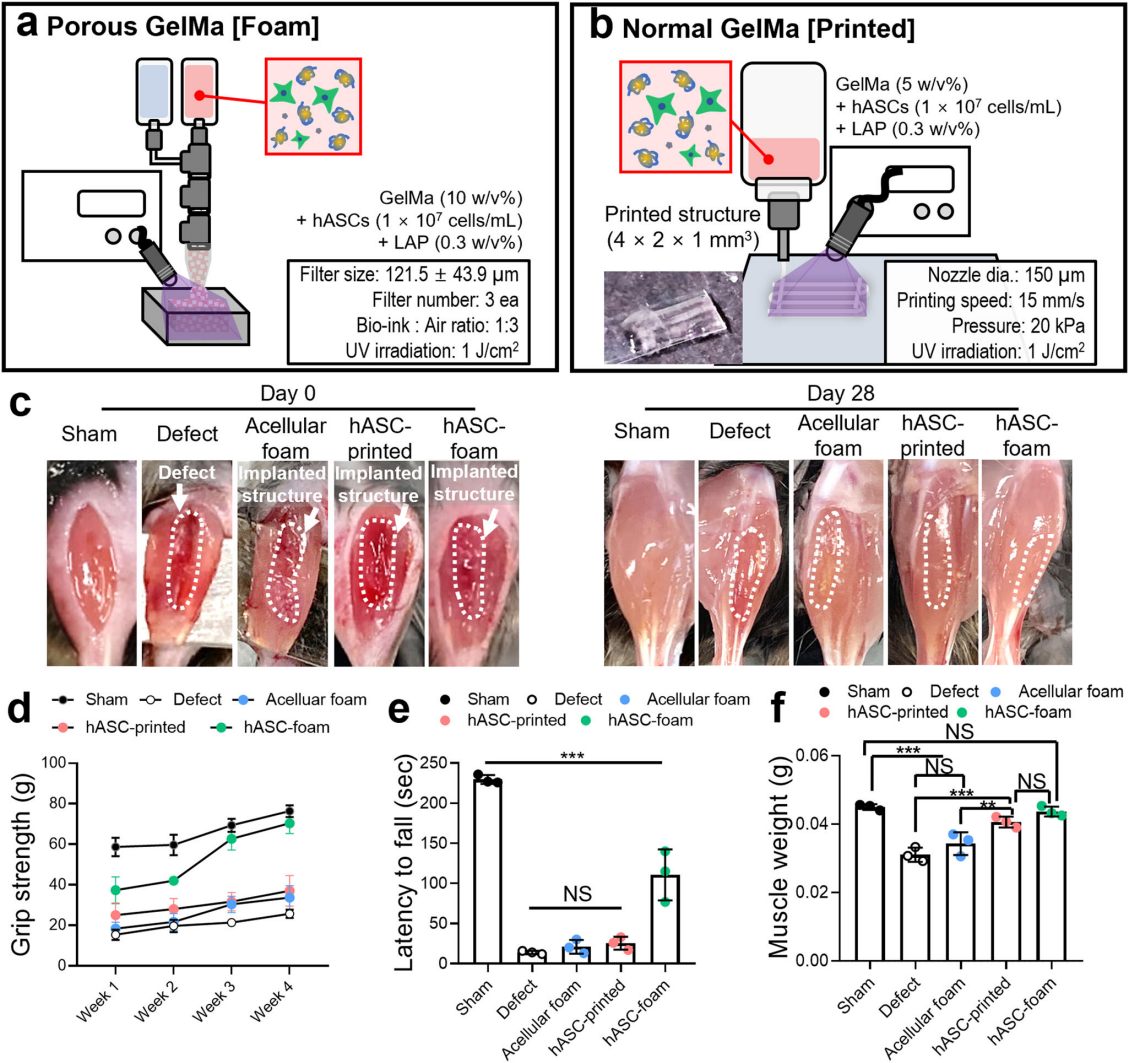
In vivo study of the porous GelMa structure. Schematics demonstrating the fabrication process and parameters of (**a**) the GelMa foam and (**b**) the normally bioprinted structure. **c** Optical images of the VML models of sham, defect, acellular foam, hASC-printed, and hASC-foam on day 0 and day 28. **d** Grip strength for 1 to 4 weeks and (**e**) latency to fall and (**f**) muscle weight at 4 weeks after implantation. All data are presented as mean ± standard deviation and the *p*-values were determined by one-way ANOVA followed by Tukey’s test (**d**, **e**, **f**) (NS = statistical nonsignificance, *p < 0.05, **p < 0.005^,^ and ***p < 0.0005).

A volumetric muscle loss (VML) defect model was prepared by removing about 40% of the tibialis anterior (TA) muscle for further verification of muscle regenerative effects on the fabricated structures, ‘Acellular foam’, ‘hASC-foam’ (Fig. 9a) and ‘hASC-printed’ (Fig. 9b). The acellular foam was fabricated via the bubble-making system using GelMa hydrogel without hASC. Previously, implantation of hASCs-laden bioconstructs has shown promising results as a VML treatment strategy owing to the myogenic lineage of hASCs^11,33,44,45^. Age-matched (Sham) and non-treated (Defect) mice were used as positive and negative controls, respectively (Fig. 9c). To evaluate muscle functionality in mice for four-weeks, grip strength (Fig. 9d) and latency to fall (Fig. 9e) were analyzed. Grip strength and fall latency were significantly higher in the hASC-foam group than in the other groups. Additionally, the muscle weight of the hASC-foam group was most similar to that of the sham group (Fig. 9f). Furthermore, the grip strength, latency to fall, and muscle weight of the mice that received the hASCs bearing foam constructs were significantly higher than the mice that received acellular foam constructs. These results indicate that the hASCs in the bioconstructs have contributed to the restoration of muscle functionality of the mice.

**Fig. 10:**
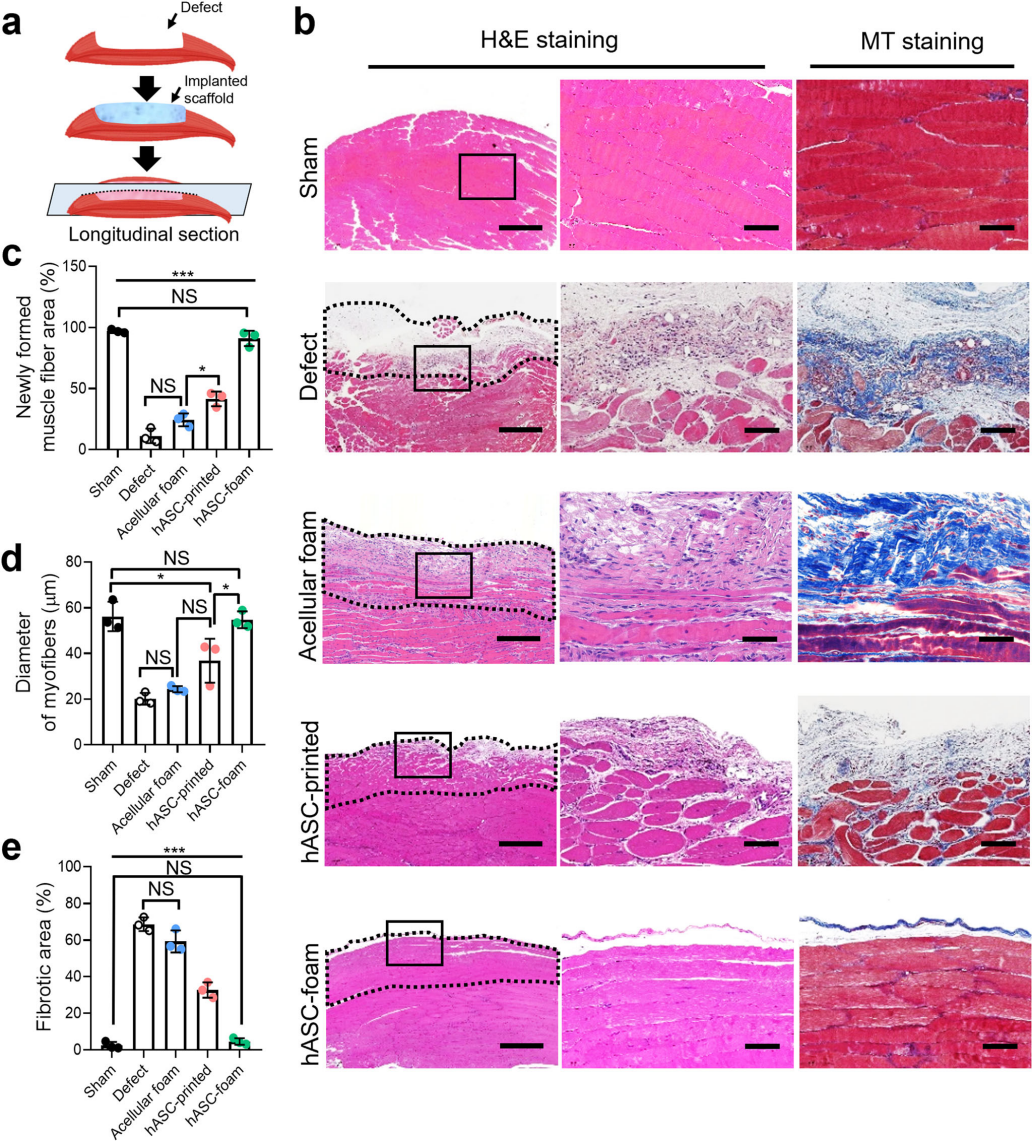
Histological staining images to observe myofiber formation. **a** Schematic of in vivo implantation and sectioning of the harvested TA muscle. **b** H&E and MT staining of implanted each group (black dotted line shows defect region). **c** The newly formed muscle fiber area, (**d**) diameter of myofibers, and (**e**) fibrotic area. All data are presented as mean ± standard deviation and the *p*-values were determined by one-way ANOVA followed by Tukey’s test (**c**, **d**, **e**) (NS = statistical nonsignificance, *p < 0.05, **p < 0.005^,^ and ***p < 0.0005). Scale bar, 500 µm (b-H&E); 50 µm (b-H&E magnified, MT).

To confirm myofiber formation, histological staining was performed with hematoxylin and eosin (H&E) and Masson’s trichrome staining (MTS) after four weeks of implantation (Fig. 10a–b). The newly generated muscle fiber area and diameter in the hASC-foam group were not significantly different from those in the sham group (Fig. 10c–d). Furthermore, the hASC-foam group showed a significantly smaller fibrotic area than the defect and hASC-printed groups (Fig. 10e). Based on these results, implantation of porous cell-laden GelMa constructs (foam group) can considerably accelerate muscle regeneration in VML defects.

**Fig. 11:**
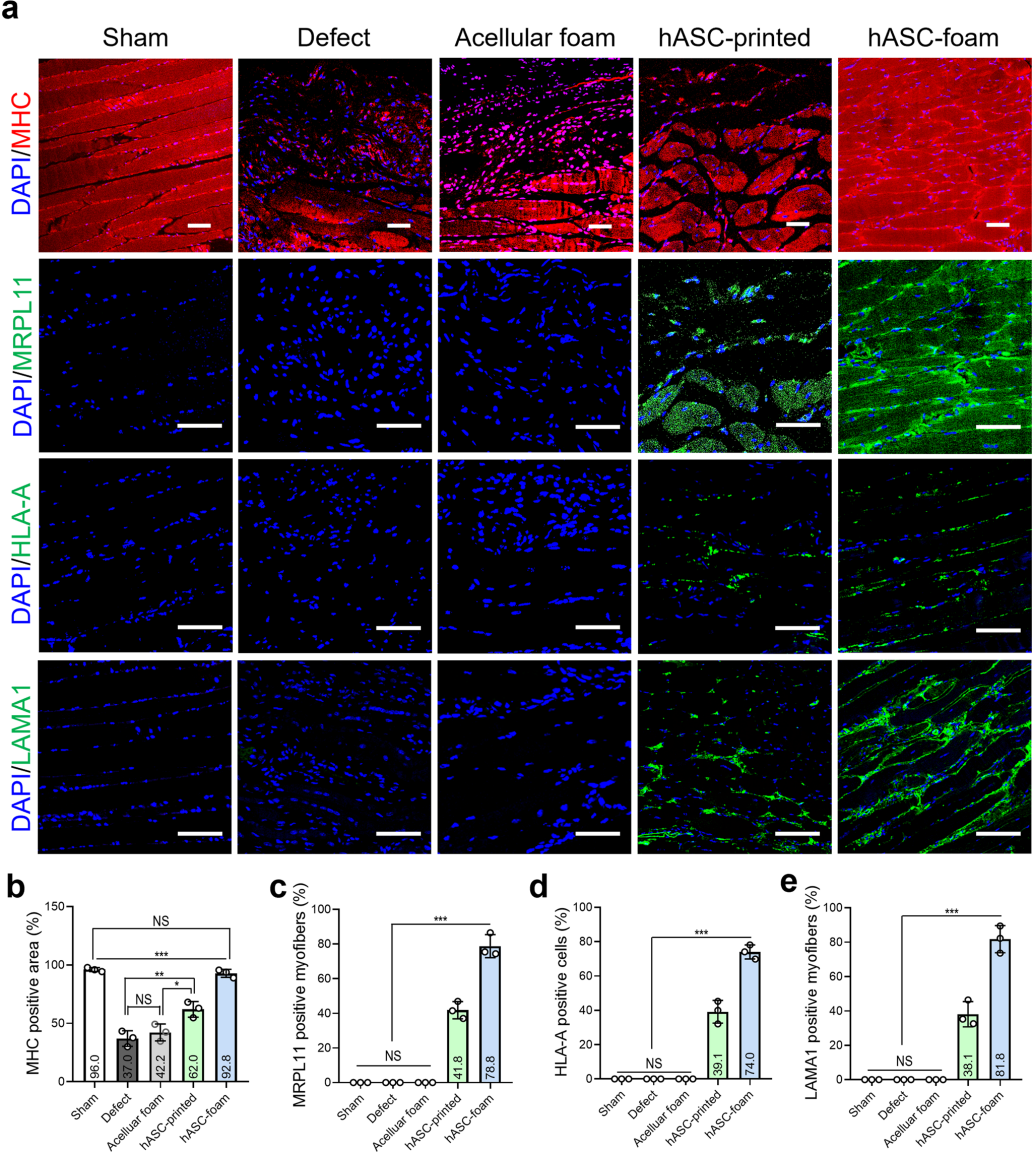
Immunofluorescence images to observe muscle regeneration. **a** Immunochemical staining images of DAPI (blue)/MHC (red) and DAPI (blue)/MRPL11 (green). **b** MHC positive area, (**c**) MRPL11 positive myofibers, (**d**) HLA-A positive cells, and (**e**) LAMA1 positive myofibers. All data are presented as mean ± standard deviation and the *p*-values were determined by one-way ANOVA followed by Tukey’s test (**b**, **c**, **d**) (NS = statistical nonsignificance, *p < 0.05, **p < 0.005, and ***p < 0.0005). Scale bar, 100 µm (**a**).

To investigate the effect of hASCs contained in porous constructs on muscle regeneration through survival and differentiation after transplantation into injured skeletal muscle, muscle sections were stained using and anti-mitochondrial ribosomal protein L11 (MRPL 11, green), anti-human leukocyte antigen (HLA, green), and anti-human laminin subunit alpha 1 (LAMA1, green) (Fig. 11a). As shown in the result, MHC, MRPL11, HLA-A, and LAMA1 were both positively expressed in the implant groups (hASC-printed and hASC-foam), indicating that the hASCs in both constructs have differentiated into myofibers, whereas sham, defect, and acellular foam groups did not express MRPL11, HLA-A, and LAMA1. Quantitative analysis showed that the MHC-positive area in the hASC-foam group was significantly higher than that in the other groups and comparable to that in the sham group (Fig. 11b). Similarly, the hASC-foam group showed a significantly higher MRPL11 positive myofibers, HLA-A positive cells, and LAMA1 positive myofibers compared with the other groups, indicating that the porous hASC-laden GelMa constructs were well differentiated into muscle fibers (Fig. 11c–e).

In this study, highly porous hASCs-laden GelMa constructs with pore size (≈350 ± 240 μm) and porosity (97%) were constructed using a handheld 3D printer supplemented with air injection and microscale mesh filters. Although the pore size and porosity were not precisely controlled, we were able to fabricate a reasonable range of macropore size and high foamability (≈93%) by manipulating various processing parameters, including mesh filter size, number, volume ratio of air and GelMa bioink, and flow rate. To examine the in vitro cellular activities of the porous cell constructs, live/dead cells and cell morphologies were measured. The fabricated porous hASC-laden GelMa constructs demonstrated that the laden cells were well lived and more actively expanded as compared to the non-porous cell-laden bioink. Moreover, the regeneration ability of the hASCs in the porous GelMa construct in the VML mouse model was compared to that of the bioprinted hASCs-laden GelMa construct. Significantly higher muscle regeneration was observed in the porous cell constructs, indicating that the handheld 3D printer can be considered a light and potential medical device for use in muscle tissue regeneration.

## Methods

### Methacrylation process of gelatin

GelMa was synthesized according to a previously described method^46^. Briefly, gelatin (300 g Bloom; Sigma Aldrich, USA) from porcine skin was dissolved in 10% w/v phosphate-buffered saline (PBS). Methacrylic anhydride (Sigma-Aldrich, USA) was added slowly while stirring at 50 °C. After 2 h, the reacted solution was dialyzed in distilled water at 40 °C for 7 days using dialysis tubes (molecular cutoff of 1000 kDa; Spectrum Labs, Inc., USA) to remove the remaining methacrylic anhydride. GelMa was lyophilized prior to use.

Lyophilized GelMa was dissolved in PBS and mixed with 0.3 mg/mL lithium phenyl-2,4,6-trimethylbenzoylphosphinate (LAP; Sigma Aldrich, USA) to obtain GelMa solutions of various concentrations (5, 10, and 15% w/v).

To evaluate the rheological properties of GelMa solutions, a rotational rheometer (Bohlin Gemini HR Nano, Malvern Instruments, UK) with a cone-and-plate geometry (1° angle, 40 mm diameter, 150 μm gap) was used to measure the complex viscosity (Pa·s). A temperature sweep (10–40 °C) of the pre-crosslinked GelMa solutions (5, 10, and 15% w/v) was conducted at a frequency of 1 Hz and 1% strain. A time sweep of GelMa solutions (5, 10, and 15% w/v) was conducted for 240 s at various UV doses using an acrylic parallel-plate geometry (diameter: 40 mm and gap: 200 μm). To provide UV dosage of 0.5, 1, 2, and 3 (J/cm^2^), UV light was directed towards the GelMa solutions from 120 to 240 s.

### Preparation of a handheld 3D printer

To fabricate porous GelMa constructs, we prepared a homemade syringe gun using a commercial clamp gun (Besto, South Korea). The syringe-holding modules were designed based on a clamp gun and manufactured using a 3D printer (Single Plus-320C; Cubicon, South Korea). The syringes contained GelMa bioink and air, respectively, and were pushed together to inject air and bioink into a three-way stopcock. Air and bioink were then passed through a filter (polyethylene mesh; AS ONE, South Korea) to form bubbles. The porous bioink was ejected through a 16 G tapered nozzle (inner diameter:1.19 mm).

### Characterization of the fabricated constructs

The fabricated porous constructs were captured using an optical microscopic (CKX41; Olympus, Japan) and a scanning electron microscopic (SEM) (SNE-3000 M; SEC Inc., South Korea). Pore sizes were calculated with 600 measurements at the bottom and top of each sample (*n* = 4). The pore size coefficient of variations was measured within the bottom and top of each sample, respectively (*n* = 4) to evaluate the distribution of pore sizes. Foamability (V_foam_/V_total_ × 100, V_total_: total volume including air bubbles and solution, and V_foam_: volume of air bubbles) was determined by measuring the foam volume portion of the fabricated construct (*n* = 8). To measure the pore sizes and foamability of the fabricated constructs, ImageJ (National Institutes of Health, Bethesda, MD, USA) was used.

### In vitro evaluations

The cell-laden porous GelMa construct [hASCs, PT-5006; Lonza, Basel, Switzerland, or C2C12, CRL-1772; ATCC, Manassas, VA, USA (1 × 10^7^ cells/mL)] was fabricated in a polydimethylsiloxane (PDMS) mold (diameter: 8 mm, height: 10 mm). Briefly, the ASCs-laden bioconstructs were cultured in a culture medium (CM) consisting of Dulbecco’s modified Eagle’s medium-low glucose (DMEM-L; Sigma-Aldrich), 10% fetal bovine serum (FBS; BioWest), and 1% penicillin-streptomycin (PS; Thermo-Fisher Scientific) at 37 °C under 5% CO2, whereas, the C2C12-laden bioconstructs were cultured in CM consisting of Dulbecco’s modified Eagle’s High-low glucose (DMEM-L; Sigma-Aldrich), 10% fetal bovine serum (FBS; BioWest), and 1% penicillin-streptomycin (PS; Thermo-Fisher Scientific). The CM was changed every two days.

To estimate cellular proliferation, the 3-(4,5-dimethylthiazol-2-yl)-2,5-diphenyltetrazolium bromide (MTT) assay was performed using the Cell Proliferation Kit I (Boehringer, Mannheim, Germany). The samples were rinsed three times with DPBS, and then incubated in MTT solution for 4 h at 37 °C to obtain purple formazan crystals by metabolically active cells. After 4 h, a solubilization solution consisting of sodium dodecyl sulfate was added to dissolve the insoluble crystals. After 24 h, the optical density (OD) of the colored solutions was measured using a microplate reader (EpochTM; BioTek, South Korea) at 570 nm.

To calculate the cell viability after the fabrication process, the samples were stained with calcein AM (0.15 mM; Invitrogen) and ethidium homodimer 1 (2 mM; Invitrogen) for 1 h at 37 °C. A Carl Zeiss confocal microscopic (LSM 700; Carl Zeiss, Germany) was used to obtain images of stained live (green) and dead (red) cells. Cell viability was estimated using ImageJ software (National Institutes of Health) by calculating the number of live cells per stained cell.

To visualize the morphologies of the cells, the cell-laden constructs were fixed with 3.7% formaldehyde (Sigma-Aldrich) and treated with 0.1% Triton X-100 (Sigma-Aldrich) to permeabilize and block nonspecific antibody binding. After washing twice with PBS, the samples were stained with dapi (1:100 in DPBS)/phalloidin (1:100 in DPBS) solution (for 1 h) at 37 °C. Confocal microscopy was used to visualize the stained nuclei (blue) and F-actin (red). ImageJ software was used to measure the F-actin area.

To conduct MHC immunofluorescent staining of the cultured cells, the samples were washed using PBS and fixated by immersing the sample in 3.7% paraformaldehyde solution for 60 min at 37 °C. Then, samples were permeabilized using 2% Triton X-100, followed by treatment using 2% bovine serum albumin (BSA; Sigma-Aldrich, USA). Then, the samples were immersed in anti-MHC primary antibody (5 μg/mL; MF20, Developmental Studies Hybridoma Bank, USA) overnight at 4 °C. Then, the samples were treated using Alexa Fluor 488 (1:50 in PBS; Invitrogen, USA) conjugated secondary antibodies for 1 h at 37 °C, then counterstained using DAPI (1:100 in DPBS). The MHC-stained cells were visualized under a confocal miscroscope and ImageJ software was used to measure the positive index of cells.

### In vivo procedure

All animal procedures were performed according to the protocol approved by the Institutional Animal Care and Research Advisory Committee at the Sungkyunkwan University School of Medicine Laboratory Animal Research Center and complied with the regulations of the Institutional Ethics Committee (SKKUIACUC2021-08-11-2). For the volumetric muscle loss (VML) injury model, ten-week-old male C57BL/6 mice (DooYeol Biotech, Inc., Seoul, Korea) were used.

Prior to operation, the mice were randomly divided into five groups (15 animals in total *n* = 3/group). Then, the mice were anesthetized using 3% isoflurane, and the left and right hind limb was depilated using a sterile blade. After that, the skin was incised approximately 4 mm, and the muscles were separated from the fascia to remove the extensor digitorum longus (EDL) and extensor hallucis longus (EHL) muscles to prevent compensatory hypertrophy during muscle regeneration. Finally, approximately 40% of the *tibialis anterior* (TA) muscle was excised and its weight was measured (Supplementary Table 1). The bioprinted constructs were implanted into the muscle-defect regions. The fascia and skin were then sutured using absorbable sutures. To control pain, 0.5 mg/kg buprenorphine was injected into the mice. Additionally, food and water were also provided normally after implantation. Five groups were performed at four-week time points considering that the robust cellular responses including angiogenesis and neurogenesis at 7~28 days for C57BL/6 mice^41,46,47^. The groups are as follows: (1) sham (age-matched control); (2) defect (no treatment); (3) hASC-printed (conventionally bioprinted hASCs-laden GelMa); (4) acellular foam (injection of porous GelMa using an in situ handheld 3D printer and (5) hASC-foam (injection of porous hASCs-laden GelMa using an in situ handheld 3D printer). After 4 weeks of implatation, the mice were euthanized via CO_2_ inhalation and secondary cervical dislocation.

### Muscle functional evaluations

The skeletal muscle functionality of mice that received various treatments was assessed by measuring hindlimb grip strength and latency to fall. Hindlimb grip strength measurements were performed using a grip strength meter (BIO-GS3, BIOSEB, FL, USA) at various intervals (1, 2, 3, and 4 weeks) after transplantation to measure maximum force^48^. Briefly, mice were allowed to grip a metal T-bar (BIO-GRIPBS Bar for mice) with their hind paws, and maximum force was measured by pulling in parallel until release^49^. Additionally, the latency to fall was evaluated by measuring the time elapsed after the mouse was placed on a rod. The maximum latency period was set at 300 s. Each experiment was repeated three times per mouse. An interval duration of 5 min was provided between the repetitions. After 4 weeks of implantation, the TA muscles of the mouse were excised and weighed.

### Histological and immunofluorescent staining

Four weeks after implantation, the TA muscles were harvested for histological and immunofluorescence staining. Briefly, the isolated TA muscles were treated with 4% paraformaldehyde at room temperature for 24 h, followed by paraffin embedding. The muscle tissues were sectioned into 5 μm thick slices. The sectioned muscle slides were stained with *hematoxylin and eosin* (H&E) and Masson’s Trichrome Staining (MTS) to assess the muscle fiber and fibrotic area. To further elucidate the muscle slides, immunofluorescent staining of MHC, MRPL11 (rabbit polyclonal antimitochondrial ribosomal protein L11, species reactivity: human, 1:1000 dilution; Abcam, Cambridge, UK), HLA-A (human leukocyte antigen, species reactivity: human, 1:100 dilution; Abcam, Cambridge, UK), and and LAMA1 (mouse monoclonal laminin subunit alpha 1, species reactivity: human, 1:500 dilution; Abnova Corporation, Taipei, Taiwan) was performed. Briefly, sections were dewaxed, rehydrated, and incubated with anti-MHC, anti-MRPL11, anti-HLA, and anti-LAMA1 antibodies overnight at 4 °C. The samples were then washed three times with PBS and incubated in either Alexa 488-conjugated anti-rabbit IgG (1:200 dilution, Invitrogen) or Alexa 488-conjugated anti-mouse IgG (1:200 dilution, Invitrogen) or Alexa 594-conjugated anti-mouse IgG (1:200 dilution, Invitrogen) for 1 h, followed by counterstaining with DAPI for 5 min. The immunofluorescent-stained samples were visualized using a confocal microscopic, and the positive areas were quantified using ImageJ software.

### Statistical analysis

All data are presented as mean ± standard deviation (SD). To conduct statistical analyses, the student’s *t*-test to compare two groups and analysis of variance (ANOVA) to compare three or more groups with Tukey’s honest significant difference post-hoc test was performed using SPSS software (SPSS, Inc., USA). Values of *p < 0.05, **p < 0.005, and ***p < 0.0005 were considered statistically significant.

### Reporting summary

Further information on research design is available in the Nature Research Reporting Summary linked to this article.

## Supplementary information

Reporting Summary

Supplemental Information

## Data Availability

The data supporting the conclusions of this study are available with the corresponding author upon reasonable request.
