# Supplementary material for: The one-step fabrication of porous hASC-laden GelMa constructs using a handheld printing system
Source: NPJ Regen Med. 2023 Jun 10;8:30. doi: 10.1038/s41536-023-00307-1 (PMC10257650; doi:10.1038/s41536-023-00307-1)
Supplement: Supplementary file 2 — Supplemental Information [file 41536_2023_307_MOESM2_ESM.pdf]

Supplementary information

The one-step fabrication of porous hASC-laden GelMa constructs using a handheld printing system

SeoYul Jo[1,†], JiUn Lee[1,†], Hyeongjin Lee[1,2], Dongryeol Ryu[3], and GeunHyung Kim[1,4,*]

[1]Department of Precision Medicine, Sungkyunkwan University School of Medicine, Suwon, Republic of Korea
[2]Department of Biotechnology and Bioinformatics, Korea University, Sejong, Republic of Korea
[3]Department of Biomedical Science and Engineering, Gwangju Institute of Science and Technology, Gwangju, Republic of Korea
[4]Department of Biophysics, Institute of Quantum Biophysics, Sungkyunkwan University, Suwon, Republic of Korea

[†]The authors contributed equally to this work.

[*]Corresponding author
Prof. GeunHyung Kim
Department of Precision Medicine, Sungkyunkwan University School of Medicine, Suwon, Republic of Korea; Tel: +82-31-290-7828; E-mail address: gkimbme@skku.edu

Supplementary Table 1. The weight of the total and removed 40% of tibialis anterior (TA) muscle in C57BL/6 mouse.

Number of mice	TA muscle weight (g)	Removed 40% of the TA muscle weight (g)
1	0.0448	0.018
2	0.0447	0.018
3	0.0420	0.017
4	0.0361	0.014
5	0.0411	0.016
6	0.0460	0.018
7	0.0440	0.018
8	0.0512	0.021
9	0.0362	0.015
10	0.0499	0.020
11	0.0393	0.016
12	0.0386	0.015
Mean ± SD	0.043 ± 0.005	0.017 ± 0.002

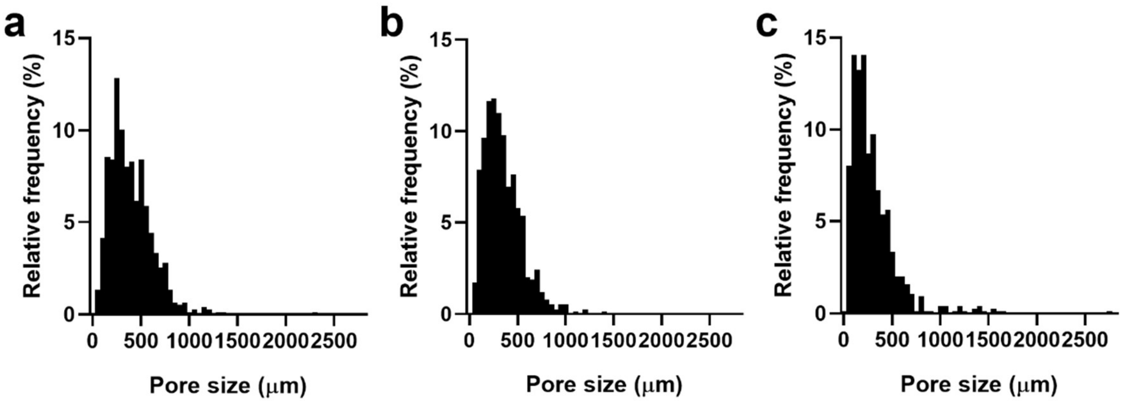

Supplementary Fig. 1 Histograms of pore sizes about various GelMa concentrations; (a) 5, (b) 10, and (c) 15% w/v.

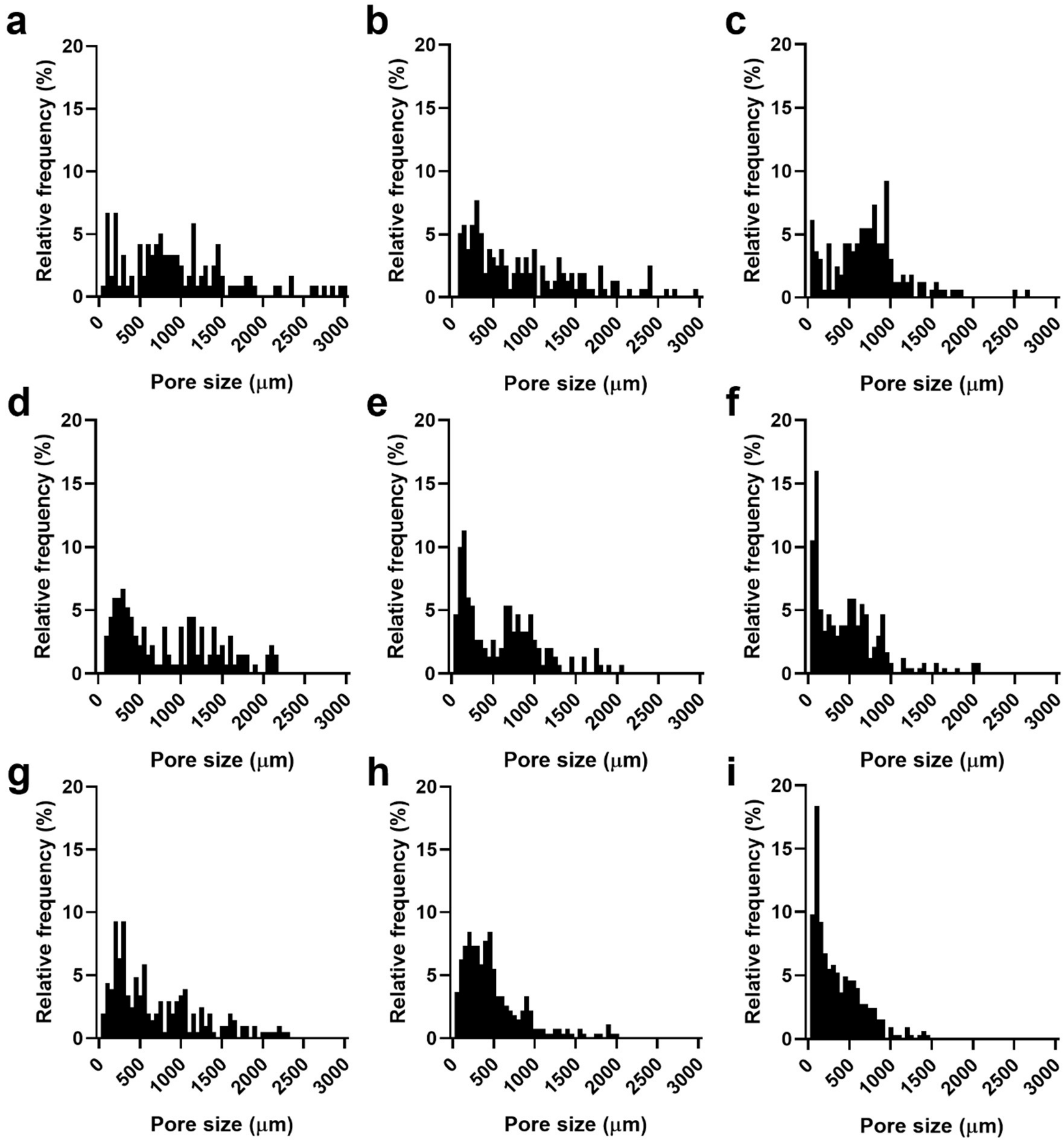

Supplementary Fig. 2 Histograms of pore sizes about various filter sizes and numbers; (a) filter size: FS-1, filter number: 1, (b) filter size: FS-2, filter number: 1, (c) filter size: FS-3, filter number: 1, (d) filter size: FS-1, filter number: 2, (e) filter size: FS-2, filter number: 2, (f) filter size: FS-3, filter number: 2, (g) filter size: FS-1, filter number: 3, (h) filter size: FS-2, filter number: 3, and (i) filter size: FS-3, filter number: 3.

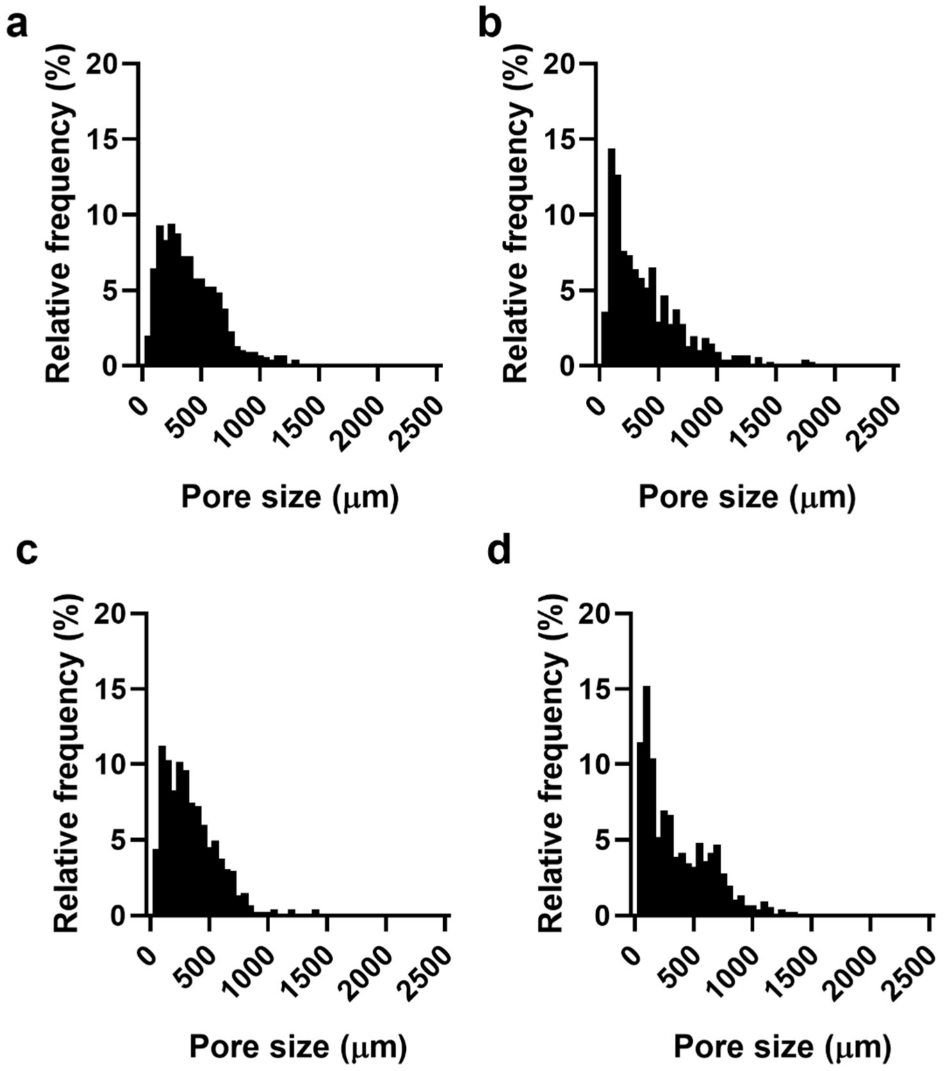

Supplementary Fig. 3 Histograms of pore sizes about various volume ratios of GelMa bio-ink and air; (a) 1:1, (b) 1:2, (c) 1:3, and (d) 1:4.

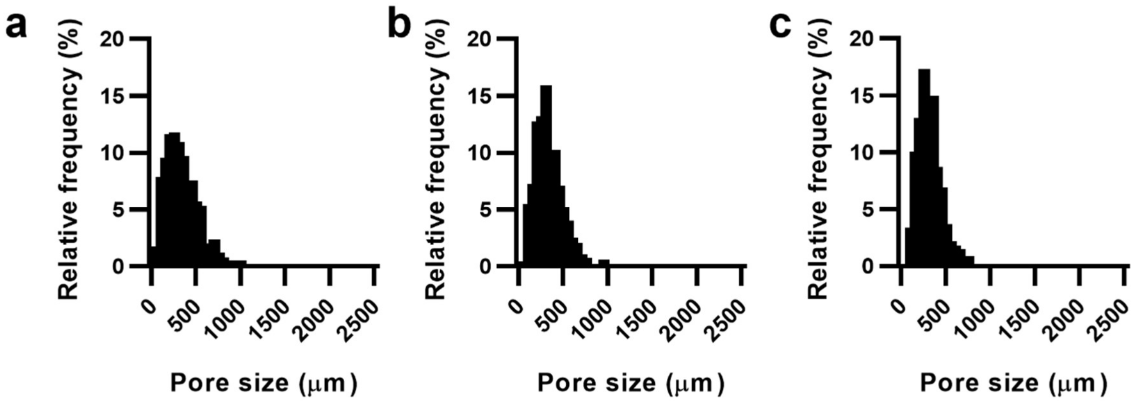

Supplementary Figure 4. Histograms of pore sizes about various flow rates; (a) 3, (b) 6, and (c) 12 mL/s.
